# X-ray Structure Elucidation of a Pt-Metalloporphyrin and Its Application for Obtaining Sensitive AuNPs-Plasmonic Hybrids Capable of Detecting Triiodide Anions

**DOI:** 10.3390/ijms20030710

**Published:** 2019-02-07

**Authors:** Eugenia Fagadar-Cosma, Anca Lascu, Sergiu Shova, Mirela-Fernanda Zaltariov, Mihaela Birdeanu, Lilia Croitor, Adriana Balan, Diana Anghel, Serban Stamatin

**Affiliations:** 1 Institute of Chemistry Timisoara of Romanian Academy, Mihai Viteazu Ave. No. 24, 300223 Timisoara, Romania; croitor.lilia@gmail.com (L.C.); dianaracanel@yahoo.com (D.A.); 2 Laboratory of Inorganic Polymers, “Petru Poni” Institute of Macromolecular Chemistry, Aleea Grigore Ghica Voda No. 41A, RO-700487 Iasi, Romania; shova@icmpp.ro (S.S.); zaltariov.mirela@icmpp.ro (M.-F.Z.); 3 National Institute for Research and Development in Electrochemistry and Condensed Matter, P. Andronescu Street, No. 1, 300224 Timisoara, Romania; mihaione2002@yahoo.com; 4 3Nano-SAE Research Center, Faculty of Physics, University of Bucharest, Atomistilor Street, No 405, 077125 Măgurele, Romania; andronie@3nanosae.org (A.B.); serban@3nanosae.org (S.S.)

**Keywords:** Pt(II) metalloporphyrin, single crystal X-ray diffraction, UV–vis spectroscopy, AFM microscopy, triiodide detection

## Abstract

The development of UV–vis spectrophotometric methods based on metalloporphyrins for fast, highly sensitive and selective anion detection, which avoids several of the practical challenges associated with other detection methods, is of tremendous importance in analytical chemistry. In this study, we focused on achieving a selective optical sensor for triiodide ion detection in traces based on a novel hybrid material comprised of Pt(II) 5,10,15,20-tetra(4-methoxy-phenyl)-porphyrin (PtTMeOPP) and gold nanoparticles (AuNPs). This sensor has high relevance in medical physiological tests. The structure of PtTMeOPP was investigated by single crystal X-ray diffraction in order to understand the metal surroundings and the molecule conformation and to assess if it qualifies as a potential sensitive material. It was proven that the Pt-porphyrin generated 1D H-bond supramolecular chains due to the weak C-H···O intermolecular hydrogen bonding. The presence of ordered voids in the crystal encouraged us to use PtTMeOPP as the sensing material for triiodide ion and to enhance its potential in a novel AuNPs/PtTMeOPP hybrid by the synergistic effects provided by the plasmonic gold nanoparticles. The spectrophotometric sensor is characterized by a detection limit of 1.5 × 10^−9^ M triiodide ion concentration and a remarkable confidence coefficient of 99.98%.

## 1. Introduction

Iodine, which is the heaviest of the halogens elements, exists as a purple–black solid that crystallizes in the orthorhombic system and can be easily sublimed. Concerning its importance to life, iodine as a micronutrient is essential in the synthesis of thyroid hormones and in the in utero neurological development of the human fetus [1]. Iodine deficiency of the mother can result in infant congenital hypothyroidism and causes mental retardation of the fetus. The estimated average requirement (EAR) of iodine in men and nonpregnant or lactating women was set by the World Health Organization (WHO) at 95 mg/day [2,3]. The recommended daily allowance by WHO and the Food and Agriculture Organization of the United Nations (FAO) for healthy adults is 150 mg/day. Nevertheless, much higher doses of iodine can be tolerated by healthy individuals without apparent ill effects.

Iodine is concentrated by the human body predominantly in the thyroid gland [4] and in other important storage tissues (especially for individuals who had their thyroid glands removed), such as the salivary glands, gastric mucosa, breast tissues, choroid plexus, ovaries and sweat glands.

It is important to add that although iodine does not easily dissociate in water and it stays in its diatomic form, it dissolves better in the present of KI because of its equilibrium with triiodide (I_3_^−^), which is 104 times more soluble in water [1]. 

On the other hand, the triiodide ion that is generated when I_2_ and I^‒^ are both in solution (see Equations (1)–(3)) is a good oxidizing agent for several reductants. Based on this property, triiodide ions are used in pharmacology for the quantitative determination of ascorbic acid and hydroquinone in drugs [5]; in controlling the environment or human health by monitoring chlorine and dissolved O_2_ in water [6]; in control of industrial processes by detection of copper and iron in ores [7]; and even as a temperature radiation detector [8,9].

I_2_ + H_2_O → HIO + H^+^ + I^−^ (1)

3HIO → 3H^+^ + IO_3_^−^ + 2I^−^ (2)

I_2_ + I^−^ → I_3_^−^ (3)

Thorough research on the evaluation of the required content of powdered supplements concluded that the body easily converts iodide to iodine under normal circumstances, with dietary supplements based on only iodide found to be equivalent to the ones consisting of both iodine and iodide [1].

Inductively coupled plasma mass spectrometry was reported to be a suitable method for the determination of iodine from saliva. Sample preparations were based on centrifugation and the direct analysis of supernatants or on microwave-induced combustion and microwave-assisted alkaline dissolution [10]. The use of microwave techniques provided better results as they were able to quantify iodine from a concentration of 0.022 μg/mL.

The detection of triiodide ion, which is the other form of iodide in aqueous media, was achieved using a selective PVC membrane electrode containing 7,16-dibenzyl-1,4,10,13-tetraoxa-7,16-diaza-cyclooctadecane in a wide concentration range (10^−1^–10^−5^ M) [11]. This electrode is also capable of quantifying ascorbic acid and hydroquinone by potentiometric titration.

On the other hand, potentiometric sensors exhibiting very high selectivity for triiodide ions over other anions, which could be used in a wide pH range of approximately 2–10, were reported using the charge-transfer complex of iodine with (4,7,13,16,21,24-hexaoxa-1,10-diazabicyclo-[8.8.8]hexacosane (cryptand 222) as an electroactive ionophore and *ortho*-nitrophenyloctyl ether as a plasticizing agent. Nernstian response to triiodide ions was reported [7] with detection limits of 6.3 × 10^−7^ and 7.9 × 10^−7^ M for cryptand 222 and its charge-transfer complex with iodine, respectively.

Several methods for the detection of halide ions have been reported [12,13] based on porphyrins as sensitive materials. In a previous published paper [14], we presented a fast, sensitive and reliable potentiometric method for the determination of iodide by using a PVC membrane containing Pt(II)-5,10,15,20-tetra(4-methoxy-phenyl)-porphyrin (PtTMeOPP) as the ionophore and different plasticizers, which were namely *o*-nitrophenyloctylether (NPOE) and dioctylphtalate (DOP). The developed potentiometric sensor has relevance in the iodide medical monitored concentration range with a detection limit of 9 × 10^−6^ M. 

There are numerous methods of quantitative determination of iodide ions, with the best results given by optical spectroscopy. The lowest detection limit (1 × 10^−12^ M) was obtained by using a water-soluble cationic Pt-porphyrin measuring the phosphorescence quenching [15].

The porphyrin macrocycle has 12 functional positions (eight *β* and four *meso*) that are potentially available to be functionalized with anchoring groups, which might be specifically tailored to be able to bind iodine [16], but the best approach to coordinate anions, such as iodide and triiodide, involves the use of appropriate metalloporphyrins.

**Scheme 1:**
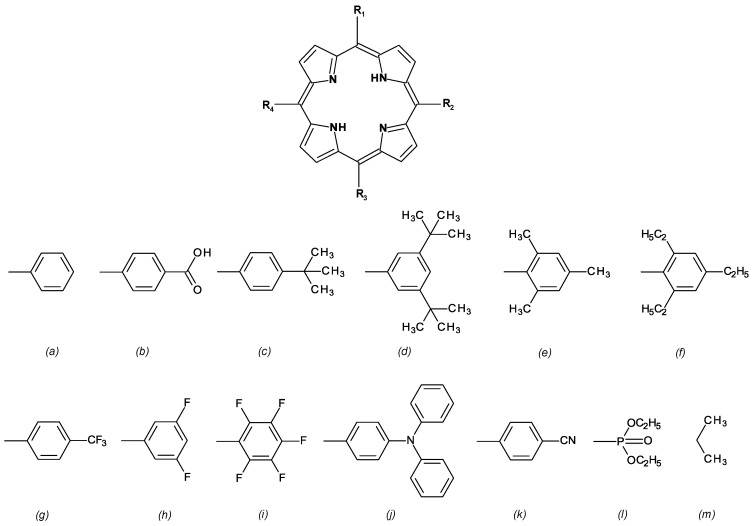
Selected substituents of Pt(II) *meso*-substituted porphyrins, whose structures were elucidated by single crystal X-ray diffraction. There were 24 A_4_-type porphyrins (R_1_=R_2_=R_3_=R_4_=H; Bu; a to j); 2 A_2_B_2_-structures (R_1_=R_3_=a; R_2_=R_4_=l and R_1_=R_3_=i; R_2_=R_4_=m) and only one A_3_B-type *meso*-substituted porphyrin (R_1_=R_2_=R_3_=a and R_4_=k).

Platinum(II) metalloporphyrins have attracted much attention due to their intense absorption in the visible region, high solubility in polymers and strong room temperature phosphorescence [17,18]. The retrieval of Cambridge Structural Database (CSD) [19] revealed 109 coordination compounds built up from platinum metal atoms and porphyrin macrocycles, with only 27 of them representing Pt(II) *meso*-substituted porphyrins. It might be emphasized that 24 results have been reported as symmetrical A_4_-type porphyrins, with R=H, butyl, phenyl and their derivatives (Scheme 1a–j), two A_2_B_2_ (Scheme 1 a/l and i/m) and only one A_3_B-type *meso*-substituted porphyrin (Scheme 1 a/k). Only a few reports mentioned the formation of 2D and 3D coordination polymers, in which the tetradentate porphyrin units are interconnected by the bridging metal ions and all of them contain the carboxy-phenyl *meso*-substituent [20,21,22].

A crystallographically well-characterized cucurbit[n]uril-porphyrin host–guest complex created between cationic tetrakis(4-pyridyl)porphyrin species of (H_6_TPyP)^4+^4Cl^−^ and tetramethyl-curcubit[6]uril (TMeQ[6]) has the capacity to adsorb iodine from solutions by the diffusion of the iodine molecules into the supramolecular framework cavities [23]. 

Together with our previous application results [24,25,26], these observations stimulated our interest to fully investigate the structure of Pt(II)-5,10,15,20-tetra(4-methoxy-phenyl)-porphyrin (PtTMeOPP) by single crystal X-ray diffraction in order to understand the metal surroundings and the molecule conformation and to assess if it qualifies as a potential sensitive material. The presence of ordered voids in the crystal encouraged us to use PtTMeOPP as the sensing material.

Spherical gold nanoparticles (AuNPs) with diameters of 10–20 nm are considered to be ideal partners for porphyrin-hybrid nanomaterials destined for sensing applications, due to their plasmonic properties and large surface-to-volume ratio. Recently, we showed an increased electrocatalytic effect on the reduction of H_2_O_2_ onto glassy carbon electrode, which was modified with ordered layers of AuNPs/Co(II) 5,10,15,20-*meso*-tetra(3-hydroxyphenyl)porphyrin [24].Other metalloporphyrins, such as (5,10,15,20-tetraphenyl)porphinato manganese(III) chloride, were demonstrated to act as a single mediator for dopamine sensing in the specific case of gold screen-printed electrodes [27]. The gold hybrid of (5,10,15,20-tetratolyl)porphinato manganese(III) chloride was capable of electrochemically detecting ascorbic acid with excellent confidence [28] and optically detecting β-carotene [25]. More complex systems based on gold nanoparticles and porphyrins impregnated in polymers were successfully applied in the electrochemical detection of glucose [29].

Based on the already proven synergistic effects of gold nanoparticles on electro-optical properties of metalloporphyrins and with the purpose to develop a better sensor with high relevance in medical physiological tests, a hybrid of PtTMeOPP with AuNPs [30] was obtained and tested as a sensitive material in the optical detection of triiodide ions. In addition, all of the materials were characterized by AFM, TEM and STEM microscopy.

## 2. Results and Discussions

### 2.1. Structure of Pt(II)-5,10,15,20-tetra(4-methoxy-phenyl)-porphyrin (PtTMeOPP) Investigated by Single Crystal X-ray Diffraction

**Figure 1:**
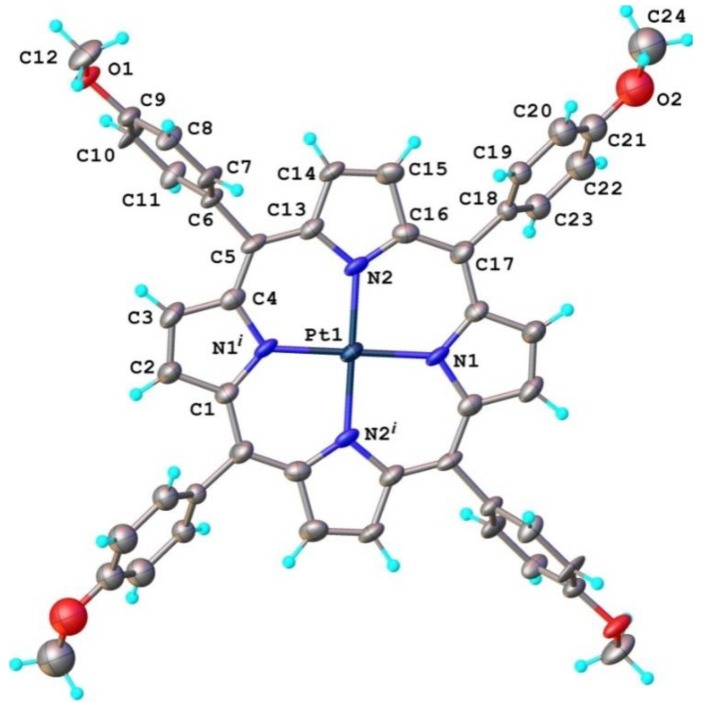
X-ray molecular structure of PtTMeOPP with atom labelling and thermal ellipsoids at 50% probability level. Symmetry code: ^i)^ 1−*x*, 2−*y*, 2−*z*.

The results of a single crystal X-ray study for compound (PtTMeOPP) are shown in Figure 1. According to X-ray crystallography, the crystal has a molecular structure that is comprised of PtTMeOPP complex units and DMF as solvate molecules in a ratio of 2:1. The Pt(II) atom occupies a special position in the center of symmetry that is coordinated by four nitrogen atoms in a square planar geometry. The metal–nitrogen bond distances of Pt1-N1 2.000(7) Å and Pt1-N2 2.017(7) Å are in good agreement with those reported for similar Pt(II) complexes [20,31,32,33,34,35]. The dihedral angles between the porphyrin nucleus and the aryl rings are 75.291(2)° (for C6>C11 ring) and 60.235(3)° (for C18>C23 ring).

**Figure 2:**
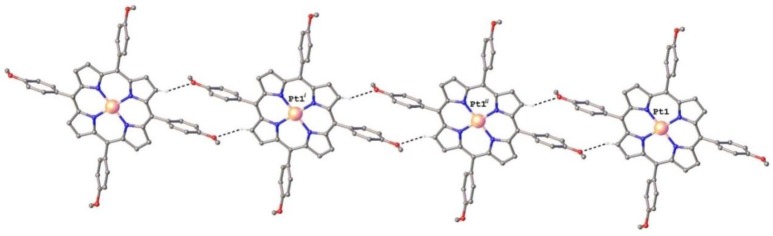
H-bonded 1D supramolecular chain in the crystal structure of (PtTMeOPP)/DMF (2:1). H-bond parameters: C14-H···O1 [C14-H 0.93 Å, H···O1 2.46 Å, C14···O1(1−*x*,1−*y*,1−*z*) 3.233(9) Å, ∠C14HO1 141.0].

**Figure 3:**
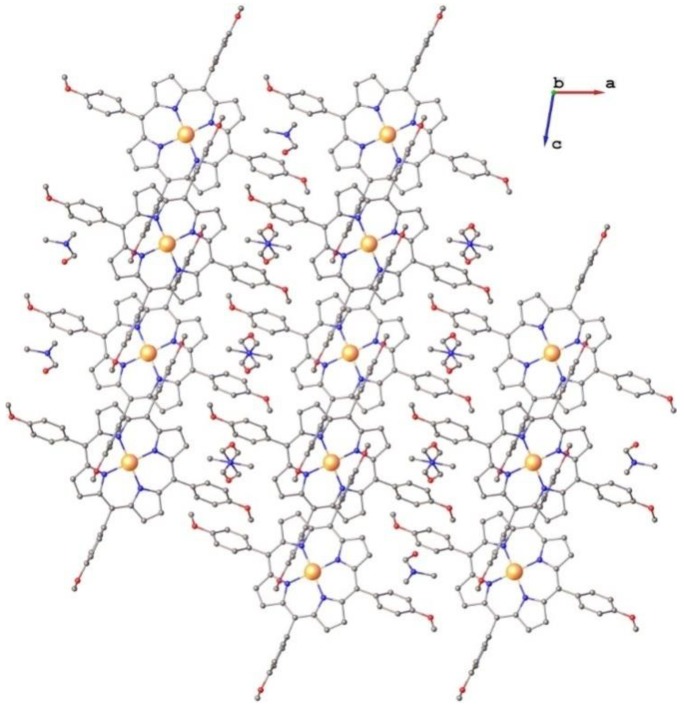
Crystal packing of (PtTMeOPP)/DMF (2:1) viewed along *b* axis.

The analysis of the crystal structure packing shows the presence of the 1D supramolecular architecture, which was generated by the weak C-H···O intermolecular hydrogen bonding of porphyrin ligand as shown in Figure 2. The volume, which is occupied by solvent molecules as calculated by PLATON [36], is 369.8 Å3 or 17.6% of the total unit cell volume for the simulated solvent-free network. A partial view of the crystal packing is shown in Figure 3.

The analysis of the Cambridge Structural Database [19] reveals 114 examples of *meso*-tetra(4-metoxyphenyl)-metalloporphyrins, but only one coordination compound of platinum has been reported so far, which is namely dibromo-(5,10,15,20-tetrakis(4-methoxyphenyl)- porphyrinato-k^4^N)-platinum(IV) grown from chloroform acetonitrile solvate [37].

### 2.2. Thermogravimetric Analysis

**Figure 4:**
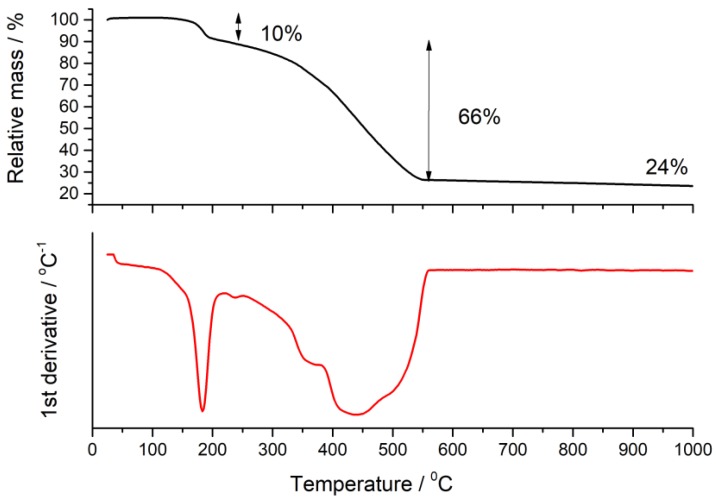
TGA pattern of PtTMeOPP.

The TGA analysis of the PtTMeOPP porphyrin (Figure 4) reveals that up to 200 °C there is a loss of weight of around 10% due to the loss of water. In this case, we presume that it is accompanied by residual small solvent molecules. In the range of temperatures from 350 to 550 °C, the carbon oxidation with a loss of around 66% weight is clearly identified. In the final stage, at around 1000 °C, the sample experiences a loss of 24% compared to its initial mass, which is a value that is very consistent with the fraction of metal weight from the molecular weight of platinum porphyrin.

### 2.3. AFM Investigations

The purpose of the topographic analysis of the surfaces performed by AFM investigation was to show if important changes occur when the AuNPs/PtTMeOPP hybrid material is formed from the initial AuNPs and PtTMeOPP and if the morphology also changes after treatment with I_3_^−^ ions, especially with regard to the self-aggregation and organization of the Pt-porphyrin.

**Figure 5:**
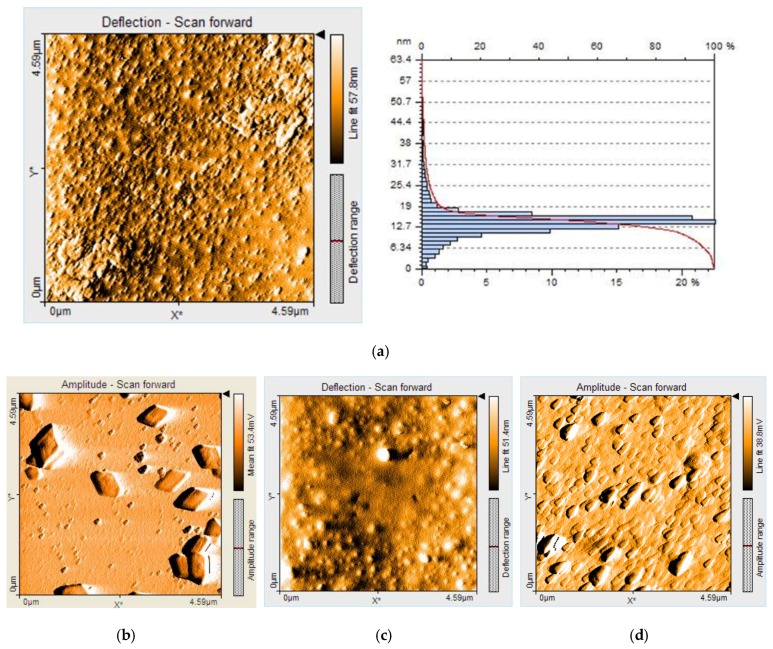
AFM shadow maps of: (**a**) AuNPs and size distribution, (**b**) PtTMeOPP, (**c**) AuNPs/PtTMeOPP hybrid material and (**d**) AuNPs/PtTMeOPP hybrid treated with I_3_^−^ ion.

**Figure 6:**
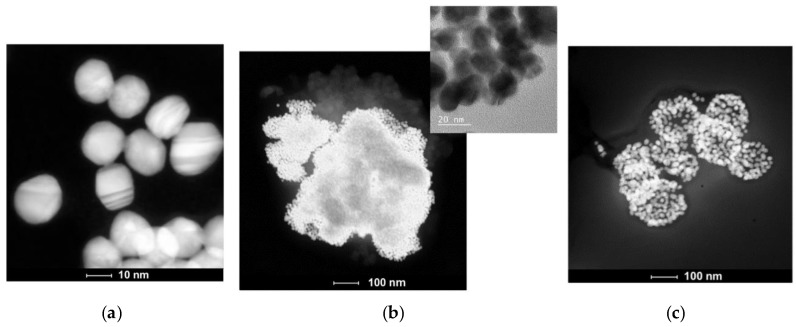
Images of: (**a**) STEM AuNPs, (**b**) STEM-TEM detail of AuNPs/PtTMeOPP hybrid material and (**c**) STEM of AuNPs/PtTMeOPP hybrid treated with I_3_^−^ ion.

The AuNPs particles, which are shown as the triangular-shaped particles in the shadow map, are uniformly oriented and have a similar size in the nano domain (Figure 5a). The size distribution of AuNPs, which was investigated by AFM, is also presented in the Figure 5a. It can easily be seen that the majority of the particles (more than 70%) have sizes around 11–15 nm, which is consistent with sizes measured from STEM images of the same AuNP nanoparticles (Figure 6a).

For the first time, the shadow map of PtTMeOPP reveals rhombohedral geometries with a greater size of approximately 200–400 nm, which can aggregate both in H- and J-types (Figure 5b). The height distribution is in the range of 15–25 nm. 

The hybrid nanomaterial shows a completely different morphology of the surface, which is characterized by straw-type large aggregates that are unevenly distributed (Figure 5c).

After treatment of the AuNPs/PtTMeOPP hybrid material with I_3_^−^ ions (Figure 5d), the architecture of the aggregates is transformed again into triangles with larger dimensions compared to the case of only AuNPs. This geometric feature is due to J-type aggregates (edge-to-edge stacked) together with H-type sandwich organization, which forms multi-layers of highly oriented triangular bricks. This type of surface is also specific to porphyrins and their hybrids [38,39] as we previously specified. It becomes clear from AFM images that after triiodide ion detection, a different structured material was generated.

### 2.4. Transmission Electron Microscopy (TEM) and Scanning Transmission Electron Microscopy (STEM) Investigations

**Figure 7:**
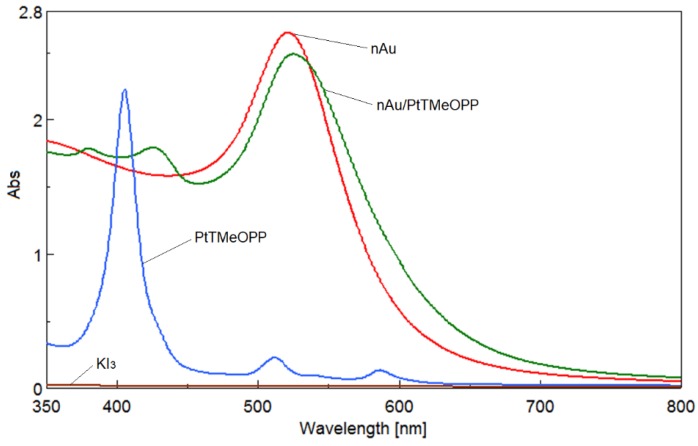
Comparative UV–vis spectra of the PtTMeOPP, AuNPs, AuNPs/PtTMeOPP hybrid and the I_3_^−^ solutions.

Our purpose was to obtain AuNPs with sizes around 15–20 nm in diameter that have a spherical or ovoidal shape and are not aggregated, which can be seen in STEM image from Figure 6a. These are considered to be the best for optical sensing due to their plasmonic behaviour (as can be further seen in Figure 7). It is well-known that highly aggregated systems jeopardize the sensing properties of any material [40] due to the fact that the aggregation of cross-linked nanoparticles is nondirective and the suspension of these aggregates is unstable due to the increased particle size and decreased repelling force.

A comparison of the images recorded for the AuNPs/PtTMeOPP hybrid material (Figure 6b) with those obtained for the same hybrid after treatment with triiodide ions (Figure 6c) highlights significant differences. The AuNPs/PtTMeOPP hybrid material is compact and not structured, displaying uniform ovoidal architectures that are slightly larger than of AuNPs (Figure 6b, TEM detail).

After exposure to the triiodide ion solution, the novel AuNPs/PtTMeOPP material became reorganized into globular clusters (Figure 6c) with greater dispersion, mimicking a dandelion plant. All these results are in agreement with AFM results and also with the spectroscopic UV–vis obtained data (see Section 2.5.2). Thus, these results provide evidence for the generation of a new intermediate compound during detection.

The UV–vis superposed spectra of Pt-metalloporphyrin, gold colloid, I_3_^−^ solution and sensitive AuNPs/PtTMeOPP hybrid material are displayed in Figure 7.

### 2.5. UV–Vis Spectrophotometric Detection of Triiodide Ion I_3_^−^ Using as Sensitive Material AuNPs/PtTMeOPP Hybrid

#### 2.5.1. Obtaining the AuNPs/PtTMeOPP Hybrid Material

**Figure 8:**
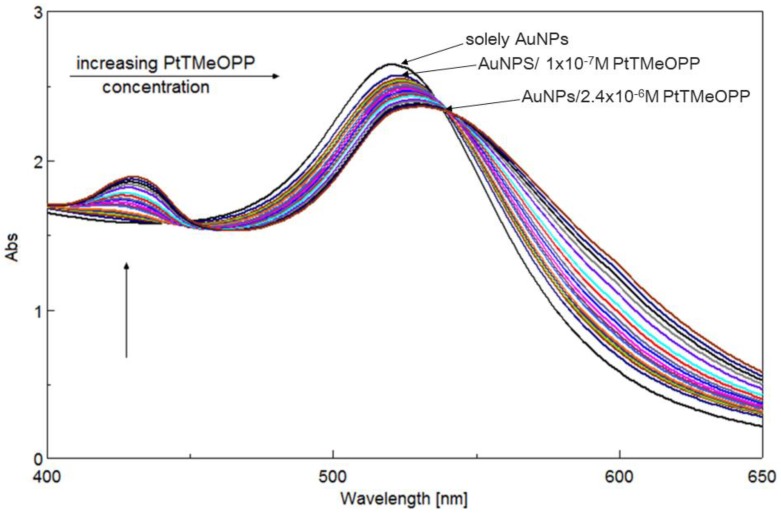
Overlapped UV–vis spectra for the formation of AuNPs/PtTMeOPP hybrid by continuously adding PtTMeOPP solution in THF to the gold colloid solution.

To a solution of gold colloid (3.6 mL, *c* = 4.5 × 10^−4^ M), portions of 60 µL of 2 × 10^−5^ M PtTMeOPP solution in THF were successively added at room temperature under vigorous stirring for 3 min. The UV–vis spectra were performed for each addition. The plasmonic band suffers both the widening of the absorption domain from 520 to 540 nm and a hypochromic effect due to the increasing concentration of PtTMeOPP. The equilibria processes that occur during the generation of the AuNPs/PtTMeOPP hybrid are proven by the existence of clearly illustrated isosbestic points at 450 and 545 nm (Figure 8).

After analyzing the optical properties (Figure 8), we used the following method to prepare the sensitive AuNPs/PtTMeOPP hybrid material: a total of 3 mL of the AuNPs solution (*c* = 4.5 × 10^−4^ M) was mixed with 0.5 mL of the PtTMeOPP solution (*c* = 2 × 10^−5^ M) in THF and stirred in ultrasonic bath to produce the hybrid material that had a violet color in the solution. As observed, the obtaining AuNPs/PtTMeOPP hybrid material is also certified by the two supplementary bands, one hipsochromically and one bathochromically located in comparison with the Soret band of the Pt-metalloporphyrin. However, both manifest a hyperchromic effect, which is consistent with literature [30].

#### 2.5.2. Detection of Triiodide Ion I_3_^−^ Using As Sensitive Material AuNPs/PtTMeOPP Hybrid

**Figure 9:**
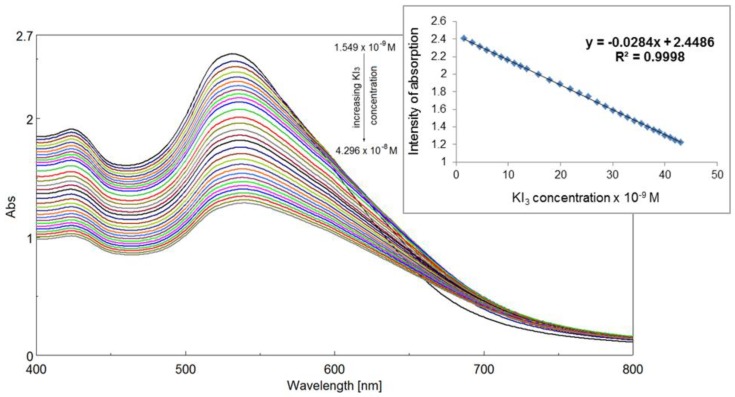
Overlapped UV–vis spectra after successive additions of I_3_^−^ solution to AuNPs/PtTMeOPP hybrid solution. In detail: the dependence of the intensity of absorption of the plasmonic band read at 520 nm on the increase in triiodide ion concentration.

The method performed for triiodide ion detection is described as follows. Well-defined portions, each consisting of 0.05 or 0.1 mL of the triiodide ion solution (*c* = 9.43 × 10^−8^ M), were added to 3 mL of AuNPs/PtTMeOPP hybrid material solution. After they were all added, the mixture was vigorously stirred at room temperature for 50 seconds and the UV–vis spectra were recorded. The corrected concentrations of triiodide ion in the mixtures are provided as follows: 1.54 × 10^−9^ M, 3.04 × 10^−9^, 4.50 × 10^−9^, 5.90 × 10^−9^, 7.26 × 10^−9^, 8.59 × 10^−9^, 9.87 × 10^−9^, 11.11 × 10^−9^, 12.32 × 10^−9^, 13.50 × 10^−9^, 15.75 × 10^−9^, 17.87 × 10^−9^, 19.89 × 10^−9^, 21.80 × 10^−9^, 23.62 × 10^−9^, 25.35 × 10^−9^, 27.00 × 10^−9^, 28.56 × 10^−9^, 30.06 × 10^−9^, 31.50 × 10^−9^, 32.86 × 10^−9^, 34.18 × 10^−9^, 35.43 × 10^−9^, 36.64 × 10^−9^, 37.80 × 10^−9^, 38.91 × 10^−9^, 39.98 × 10^−9^, 41.00 × 10^−9^, 42.00 × 10^−9^ and 42.95 × 10^−9^. The intensity of absorption function of these concentrations is shown in Figure 9. The sensing process is based on the formation of an intermediate compound, which was proven by the change in the shape of the hybrid and by the presence of the isosbestic point around 720 nm. Besides, a blind test to see the influence of only dilution on the UV–vis behavior of the AuNPs-Pt(II)porphyrin hybrid, which showed a limited and chaotic response, additionally proves that the proposed system for detection of triiodide ion is not due to dilution.

When the I_3_^−^ concentration was in the range from 1.55 × 10^−9^ to 4.3 × 10^−8^ M, the dependence between the intensity of absorption of the plasmonic band read at 520 nm and the increasing concentration of triiodide ion was perfectly linear, which was characterized by an excellent correlation coefficient of 99.98% (Figure 9).

**Figure 10:**
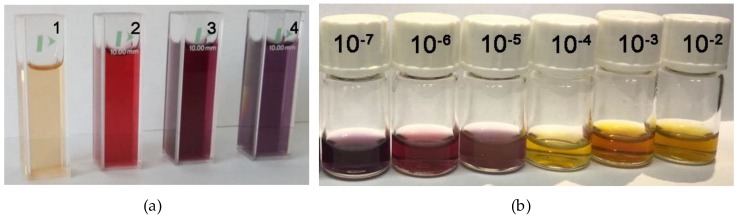
(**a**) Color changes for: PtTMeOPP (1); AuNPs (2); AuNPs/PtTMeOPP hybrid (3); AuNPs/PtTMeOPP hybrid treated with I_3_^−^ ion (4); (**b**) color change with an increase in I_3_^−^ ion concentration from 10^−7^ to 10^−2^ M.

Both the processes of AuNPs/PtTMeOPP hybrid generation and triiodide I_3_^‒^ ion detection are accompanied by a significant change in color that encouraged us to further investigate their potential as colorimetric sensors (Figure 10a).

The spectacular changes in color (Figure 10b), might be explained by the already proven nonlinear absorption coefficients of triiodide ion observed at the formation of clusters between I_3_^−^ and water molecules [9].

#### 2.5.3. Detection of Triiodide ion I_3_^−^ Using As Sensitive Material AuNPs/PtTMeOPP Hybrid in Phosphate Buffer

Our final purpose will be to use this method to detect triiodide ions in medical/biological samples. As these samples usually contain a high level of salt, which may disturb the AuNPs and cause aggregation, we also studied the behavior of AuNPs/PtTMeOPP hybrid in a phosphate-buffered solution.

**Figure 11:**
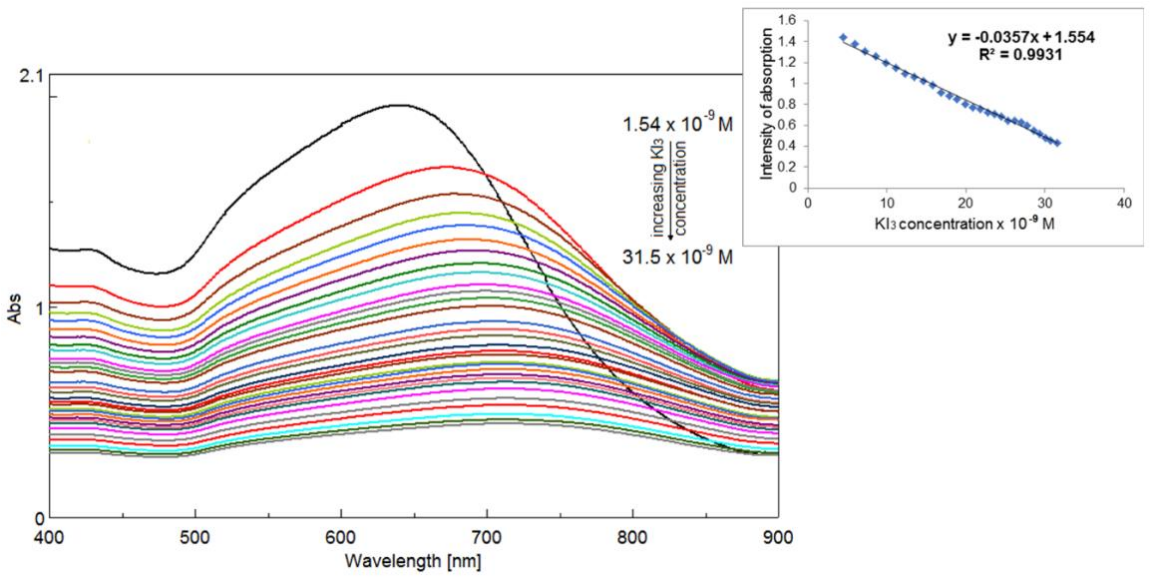
The overlapped UV–vis spectra recorded after treating phosphate-buffered AuNPs/PtTMeOPP hybrid with I_3_^−^ increased concentrations from 1.54 × 10^−9^ to 3.15 × 10^−8^ M. Dependence of the intensity of absorption read at 674 nm and the I_3_^−^ concentration.

The buffered solution was obtained as follows. To 3.5 mL of the AuNPs/PtTMeOPP hybrid (pH = 6.65), 6 drops of phosphate buffer (HI 70007 pH = 7.01) from Hanna Instruments were added to reach a pH of 7.01. To 3 mL of the AuNPs/PtTMeOPP hybrid that was buffered to a pH of 7.01, 0.05-mL portions of I_3_^−^ solution in water (*c* = 9.8 × 10^−8^ M) were added successively. Each mixture was stirred for 50 sec and the UV–vis spectra were recorded. The overlapped UV–vis spectra that were recorded after treating the phosphate-buffered AuNPs/PtTMeOPP hybrid with I_3_^−^ solution are presented in Figure 11. As shown in Figure 11, the position of the peak in phosphate buffered AuNPs/PtTMeOPP hybrid solution became bathochromically shifted to 674 nm. It is well-known that the addition of triiodide anion to several types of phosphates [41] generates better absorption phenomena, resulting in the widening of the absorption bands. This is the main reason why the iodine/triiodide system is also added in the formulation of dye based photovoltaic cells. Nevertheless, the detection occurs in the same way as before, with very good confidence index of 99.31%. The dependence of the intensity of absorption read at 674 nm and the I_3_^−^ concentration is represented in Figure 11.

From this experiment, it is clear that our proposed method can be used to detect triiodide ions with very good confidence in salted biological systems.

#### 2.5.4. Interference Study

**Figure 12:**
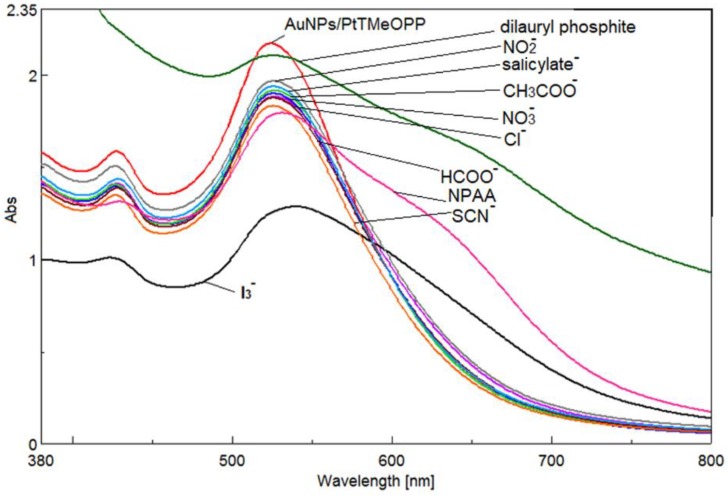
Overlapped UV–vis spectra representing the influence of diverse mono-anions upon the AuNPs/PtTMeOPP hybrid plasmon at concentrations that are 1000-fold higher than I_3_^−^.

**Figure 13:**
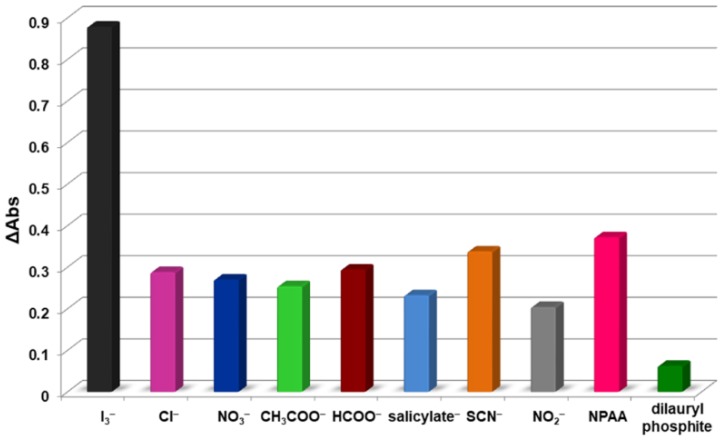
The graphical representation of the differences in absorption intensity produced by several ions interfering with the AuNPs/PtTMeOPP hybrid at concentrations that are 1000-fold higher than I_3_^−^.

The effect of common interfering ions, such as Cl^−^, NO_3_^−^, NO_2_^−^, SCN^−^, HCOO^−^, CH_3_COO^−^ and salicylate anion, were assessed and presented in Figure 12 and Figure 13.

A total of 0.5 mL of mono-anionic salt solutions in water (KCl, KNO_3_, NaNO_2_, NaSCN, HCOONa, CH_3_COONa and sodium salicylate) with a determined concentration of *c* = 1.35 × 10^−5^ M was added to 3-mL fresh portions of the hybrid solution. Besides, due to the fact that this method is destined to be used in biological tests, two other interferences, a lipid and an amino-acid, which were namely dilaurylphosphite and *N*-phenylantranilic acid (NPAA), were tested at the same concentration of 10^−5^ M and dissolved in toluene. In the case of dilauryl phosphite, the changes in the shape of the spectrum are due to the hydrophobic interactions between dilauryl phosphite, which is a surfactant agent and Pt-porphyrin component in the hybrid that can be also considered as a surfactant agent, generating H and J-type aggregates [42,43].

This concentration represents a 1000-fold increase compared to the KI_3_ detected concentration domain. From Figure 12 and Figure 13, it can be concluded that the proposed UV–vis spectrophotometric method possessed good selectivity toward I_3_^−^ determination.

**Figure 14:**
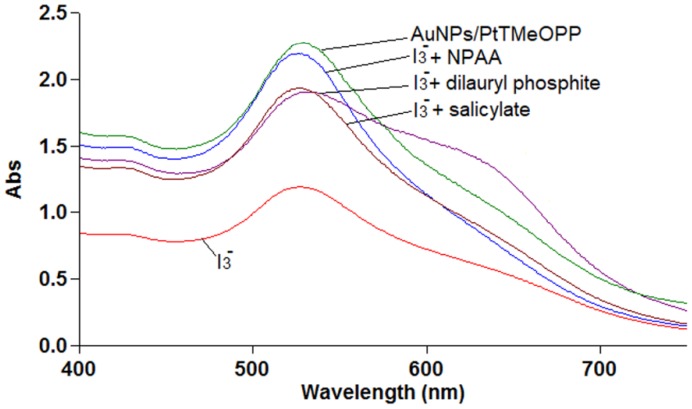
The graphical representation of the changes in absorption intensity of AuNPs/PtTMeOPP hybrid, produced by several mixtures of biological anions with triiodide anion.

In addition, the response of the hybrid material to triiodide ions after its reaction with a few potential biological interfering species was measured in order to test if the material is blocked by other anions, as described in the published paper [44]. The selected mixtures were triiodide-dilauryl phosphite, triiodide-NPAA and triiodide-salicylate, which were realized by stirring together 3-mL fresh portions from the hybrid solution with 0.2 mL of the triiodide solution with a concentration of 10^−8^ M and 0.2 mL of each interfering ion with a concentration of 10^−6^ M. As shown in Figure 14, the AuNPs/PtTMeOPP hybrid material is not blocked by amino acids, such as NPAA; lipids, such as dilauryl phosphite and not even salicylate anion, although each interference ion concentration in the mixture was more than 100 times higher than that of the triiodide anion.

#### 2.5.5. Preliminary Mechanism Investigations for I_3_^−^ Detection

**Figure 15:**
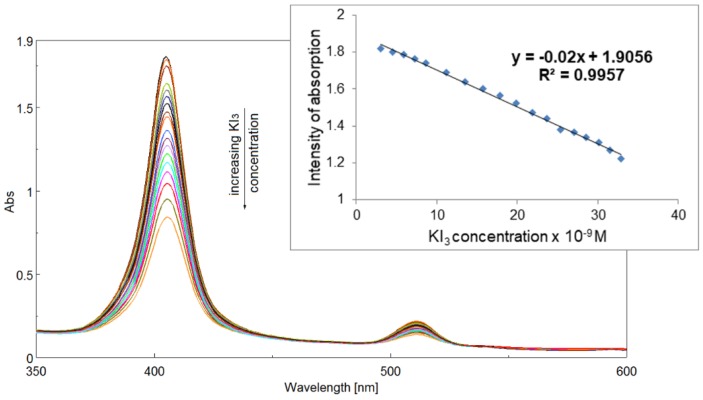
Overlapped UV–vis spectra after successive additions of I_3_^−^ solution to PtTMeOPP solution in THF. In detail: dependence between the intensity of absorption read at 405 nm and the I_3_^−^ concentration.

Based on the excellent results, we wanted to see which components of the hybrids are responsible for the triiodide ion detection. After that, we comparatively tested AuNPs alone and the Pt(II)-porphyrin alone (Figure 15).

In case of only using the sensitive material AuNPs colloid, it can be concluded that the I_3_^−^ solution produces only a dilution phenomenon with no other changes in optical behavior. This behavior can be explained by the well-known negatively charged surface of AuNPs that prevents enough proximity for the triiodide anions to be detected [45].

The second case was involved using PtTMeOPP alone as the sensitive material, following the same procedure for the spectrophotometric detection. In this case, the detection of triiodide ion is characterized by a narrow range of concentration domain and a decrease in the confidence coefficient from 99.98 to 99.57%. Besides, as shown in Figure 15, the sensitivity is significantly diminished.

Thus, from the comparatively performed studies, we can conclude that the best detection in terms of concentration range, detection limit and sensitivity is provided by AuNPs/PtTMeOPP hybrid. Regarding the contribution of the components, only the porphyrin is responsible for the chemical detection of the triiodide ion, but the AuNPs component of the hybrid contributes to better accuracy, enhanced sensitivity and lower limit of detection.

## 3. Materials and Methods 

### 3.1. Chemicals

Anisaldehyde was acquired from Roth (Karlsruhe Rheinhafen, Germany) while propionic acid and propionic anhydride were obtained from Merck (Darmstadt, Germany). Chloroauric acid tetrahydrate was purchased from Roth (Karlsruhe, Germany) while sodium citrate and THF were acquired from Merck (Darmstadt, Germany). The phosphate buffer (HI 70007 with pH = 7.01) was acquired from Hanna Instruments Inc. (Highland Industrial Park, Woonsocket, RI, USA).

The I_3_^−^ complex anion was prepared by mixing solutions of potassium iodide and molecular iodine. Solutions with a known concentration of I_3_^−^ were obtained by selecting the concentrations of I_2_ and I^−^ to enable the shift to the right of the reaction (Equation (3)) [9].

The porphyrin base, 5,10,15,20-tetra(4-methoxy-phenyl)-porphyrin, was obtained in our laboratory by Adler method [46].

### 3.2. Synthesis

#### 3.2.1. Synthesis of Pt(II) 5,10,15,20-tetra(4-methoxy-phenyl)-porphyrin

The synthesis of PtTMeOPP was realized as previously reported [14] by classical metalation reaction of 5,10,15,20-tetra(4-methoxy-phenyl)-porphyrin base with excess water soluble complex PtCl_2_(PhCN)_2_ using a large excess (ten times more) of CH_3_COONa × 3H_2_O in order to capture the chloride ion and characterized by UV–vis, FT-IR and ^1^H-NMR spectroscopy. This avoids major acidification of medium and decomposition of complexes. This simple change offers better yield in a significantly shorter time of synthesis.

#### 3.2.2. Single Crystal Preparation of Pt(II) 5,10,15,20-tetra(4-methoxy-phenyl)-porphyrin (PtTMeOPP)/DMF (2:1)

PtTMeOPP was recrystallized by using the CH_3_OH/CHCl_3_/DMF solvent mixture. About 5 mg of PtTMeOPP was dissolved in the above-mentioned solvent mixture that contained 1 mL of CH_3_OH, 0.5 mL of CHCl_3_ and 5–6 drops of DMF. The mixture was transferred into a 57 × 16 mm glass tube. The slow diffusion of 1 mL of diethyl ether into the solvent mixture of PtTMeOPP over a period of 7 days allowed us to obtain rhomboid red brown X-ray-quality single crystals of PtTMeOPP/DMF (2:1). The crystals were filtered off, washed with methanol/diethyl ether 2:1 and dried in air at room temperature. 

#### 3.2.3. Synthesis of AuNPs

The method for the synthesis of the gold colloid can be considered to belong to green chemistry and was adapted from literature [26,47]. A total of 0.025 g of HAuCl_4_·3H_2_O (6.35 × 10^−5^ mol) was dissolved in 83 mL of distilled water and brought to reflux in a 150-mL round bottomed flask. After this, 8.75 mL (1 wt %) solution of trisodium citrate (0.087 g, 2.97 × 10^−4^ mol) in distilled water was added once. The mixture was stirred vigorously and refluxed until the initially yellow solution turned black and then dark red. The molar ratio of gold salt:sodium citrate was 1:5.

### 3.3. X-ray Crystallography

X-ray diffraction measurements were obtained using an Oxford-Diffraction XCALIBUR E CCD diffractometer (Abingdon, Oxfordshire, United Kingdom) equipped with graphite-monochromated MoKα radiation. Single crystals were positioned at 40 mm from the detector and 235 frames were measured for 50 s over 1° scan width. The unit cell determination and data integration were carried out using the CrysAlis package of Oxford Diffraction [48]. The structures were solved by direct methods using Olex2 [49] software with the SHELXS structure solution program and refined by full-matrix least-squares on *F*^2^ with SHELXL-2015 [50] using an anisotropic model for non-hydrogen atoms. All H atoms were introduced in idealized positions (d_CH_ = 0.96 Å) using the riding model. The co-crystallized DMF molecule and one of the 4-methoxy-phenyl branches became disordered over two positions (50/50% and 60/40% occupancy, respectively). The molecular plots were obtained using the Olex2 program. The solvent accessible voids (SAVs) were calculated using PLATON [36]. The crystallographic data and refinement details are shown in Table 1, while bond lengths are summarized in Table S1. Supplementary data is also containing checkCIF/PLATON report. CCDC 1883498 contains the supplementary crystallographic data for this contribution. These data can be obtained free of charge via www.ccdc.cam.ac.uk/conts/retrieving.html (or from the Cambridge Crystallographic Data Centre, 12 Union Road, Cambridge CB2 1EZ, UK; fax: (+44)-1223-336-033; or deposit@ccdc.ca.ac.uk).

### 3.4. UV–Visible Spectral Studies

UV–visible spectroscopy was investigated on a V-650—JASCO spectrometer (Pfungstadt, Germany) using 1-cm wide quartz cuvettes. 

### 3.5. AFM, STEM, TEM Imaging

Atomic force microscopy (AFM) images were obtained on Nanosurf^®^EasyScan 2 Advanced Research AFM microscope (Liestal, Switzerland) in non-contact mode. The samples were deposited from solvent mixtures (THF/water) onto pure silica plates.

STEM images were recorded on a Titan G2 80-200 TEM/STEM microscope (FEI Company, Eindhoven, The Netherlands). The probes were prepared on TEM copper grids (200 mesh), which were coated with a carbon film. The analyzed compounds and the materials were drop-casted from THF-water mixtures onto the grids and the images were registered at 200 kV using TEM Imaging & Analysis v. 4.7 software.

### 3.6. Thermogravimetric Analysis

TGA was conducted on Mettler Toledo STARe System (TGA/SDTA Mettler Toledo, Schwerzenbach, Switzerland) in air. A weighed crystalline sample was placed into an alumina crucible and heated in a static atmosphere of air at a rate of 10 K/min in the temperature range of 298–1273 K (25–1000 °C).

## 4. Conclusions

The planar structure of Pt(II)-5,10,15,20-tetra(4-methoxy-phenyl)-porphyrin (PtTMeOPP) was elucidated by single crystal X-ray diffraction. The crystal structure packing shows the presence of the 1D supramolecular architecture generated due to weak C-H···O intermolecular hydrogen bonding and π-π stacking interactions between adjacent phenyl rings. The presence of ordered voids in the crystal encouraged us to obtain and use a hybrid of PtTMeOPP with AuNPs. Furthermore, we tested it as an optical sensing material for triiodide ion detection in traces, which is an investigation that is highly relevant in medical physiological tests. A highly sensitive and selective optical sensor for the detection of I_3_^−^ anions was developed, which had a detection limit of 1.5 × 10^−9^ M concentration and a confidence coefficient of 99.98%. The mechanism of detection might be explained both by the planarity and hydrophobicity of the Pt(II)-porphyrin, which offers enough room to intercalate I_3_^−^ anions between the π-π stacking 1D supramolecular chains. This takes advantage of the available voids and the well-known ability of the Pt metal to bind new anionic ligands.

## Figures and Tables

**Table 1:** Crystal data and details of data collection.

Compound	(PtTMeOPP)/DMF (2:1)
empirical formula	C_49.5_H_40_N_4.5_O_4.5_Pt
Fw	964.95
*T* [K]	180
Crystal system	monoclinic
space group	*P*2_1_/*c*
*a* [Å]	14.1668(15)
*b* [Å]	9.6654(5)
*c* [Å]	15.5836(10)
*β* [°]	99.744(8)
*V* [Å^3^]	2103.0(3)
*Z*	2
*ρ*_alcd_ [g·cm^−3^]	1.524
*μ* [mm^−1^]	3.388
Crystal size [mm]	0.15 × 0.1 × 0.02
2Θ range	4.98 to 50.052
Reflections collected	8329
Independent reflections	3712 [*R*_int_ = 0.0544]
Data/restraints/parameters	3712/9/241
*R*_1_^[a]^	0.0495
*wR*_2_^[b]^	0.1214
GOF ^[c]^	1.029
Largest diff. peak/hole/e Å^−3^	1.33/−0.82

^a^*R*_1_ = Σ||*F*_o_| − |*F*_c_||/Σ|*F*_o_|. ^b^ *wR*_2_ = {Σ[*w*(*F*_o_^2^ − *F*_c_^2^)^2^]/Σ[*w*(*F*_o_^2^)^2^]}^1/2^. ^c^ GOF = {Σ[*w*(*F*_o_^2^ − *F*_c_^2^)^2^]/(*n* − *p*)}^1/2^, where *n* is the number of reflections and *p* is the total number of parameters refined.

## Supplementary Materials

Supplementary materials can be found at http://www.mdpi.com/1422-0067/20/3/710/s1.

## Abbreviations

EAR	The estimated average requirement
WHO	World Health Organization
FAO	Food and Agriculture Organization of the United Nations
PVC	Polyvinylchloride
NPOE	*O*-Nitrophenyloctylether
DOP	Dioctylphtalate
CSD	Cambridge Structural Database
PtTMeOPP	Pt(II) 5,10,15,20-tetra(4-methoxy-phenyl)-porphyrin
AuNPs	Gold nanoparticles
AFM	Atomic force microscopy
DMF	Dimethylformamide
GOF	Goodness of fit
TGA	Thermogravimetric analysis
THF	Tetrahydrofuran
NPAA	*N*-phenylantranilic acid
