# Supplementary material for: X-ray Structure Elucidation of a Pt-Metalloporphyrin and Its Application for Obtaining Sensitive AuNPs-Plasmonic Hybrids Capable of Detecting Triiodide Anions
Source: Int J Mol Sci. 2019 Feb 7;20(3):710. doi: 10.3390/ijms20030710 (PMC6387073; doi:10.3390/ijms20030710)

checkCIF/PLATON report

Structure factors have been supplied for datablock(s) shi_3848_mz

THIS REPORT IS FOR GUIDANCE ONLY. IF USED AS PART OF A REVIEW PROCEDURE FOR PUBLICATION, IT SHOULD NOT REPLACE THE EXPERTISE OF AN EXPERIENCED CRYSTALLOGRAPHIC REFEREE.

No syntax errors found. CIF dictionary Interpreting this report

Datablock: shi_3848_mz

Bond precision: C-C = 0.0118 A Wavelength=0.71073

Cell:	a=14.1668(15)	b=9.6654(5)	c=15.5836(10)
	alpha=90	beta=99.744(8)	gamma=90
Temperature:	180 K		

	Calculated	Reported
Volume	2103.0(3)	2103.0(3)
Space group	P 21/c	P 1 21/c 1
Hall group	-P 2ybc	-P 2ybc
Moiety formula	2(C48 H36 N4 O4 Pt), C3 H8 N O	C48 H36 N4 O4 Pt, 0.25(C6 H16 N2 O2)
Sum formula	C99 H80 N9 O9 Pt2	C49.50 H40 N4.50 O4.50 Pt
Mr	1929.88	964.95
Dx,g cm-3	1.524	1.524
Z	1	2
Mu (mm-1)	3.388	3.388
F000	965.0	965.0
F000’	961.99	
h,k,lmax	16,11,18	16,11,18
Nref	3727	3705
Tmin,Tmax	0.673,0.934	0.982,1.000
Tmin’	0.596	

Correction method= # Reported T Limits: Tmin=0.982 Tmax=1.000
AbsCorr = MULTI-SCAN

Data completeness= 0.994 Theta(max)= 25.026

R(reflections)= 0.0488(2415) wR2(reflections)= 0.1187(3705)

S = 1.000 Npar= 240

The following ALERTS were generated. Each ALERT has the format
test-name_ALERT_alert-type_alert-level.
Click on the hyperlinks for more details of the test.

Alert level A

```
PLAT770_ALERT_2_A Suspect C-H Bond in CIF:  C26      --H27A      .       1.44 Ang.
PLAT772_ALERT_2_A Suspect O-H Bond in CIF: O3       --H27D      ..      1.52 Ang.
```

Alert level B

```
PLAT201_ALERT_2_B Isotropic non-H Atoms in Main Residue(s) .......         8 Report
PLAT772_ALERT_2_B Suspect O-H Bond in CIF: O3       --H27B      ..      1.49 Ang.
```

Alert level C

```
PLAT213_ALERT_2_C Atom C8              has ADP max/min Ratio .....       3.6 prolat
PLAT220_ALERT_2_C Non-Solvent  Resd 1  C   Ueq(max)/Ueq(min) Range       3.7 Ratio
PLAT250_ALERT_2_C Large U3/U1 Ratio for Average U(i,j) Tensor ....       2.6 Note
PLAT342_ALERT_3_C Low Bond Precision on  C-C Bonds ...............   0.01182 Ang.
PLAT906_ALERT_3_C Large K Value in the Analysis of Variance ......    32.366 Check
PLAT906_ALERT_3_C Large K Value in the Analysis of Variance ......     4.672 Check
PLAT911_ALERT_3_C Missing FCF Refl Between Thmin & STh/L=    0.595        21 Report
PLAT973_ALERT_2_C Check Calcd Positive Resid. Density on       Pt1      1.02 eA-3
PLAT975_ALERT_2_C Check Calcd Resid. Dens.  0.79A   From O1             0.43 eA-3
PLAT977_ALERT_2_C Check Negative Difference Density on H27C            -0.41 eA-3
```

Alert level G

```
PLAT002_ALERT_2_G Number of Distance or Angle Restraints on AtSite        13 Note
PLAT003_ALERT_2_G Number of Uiso or Uij Restrained non-H Atoms ...         5 Report
PLAT012_ALERT_1_G No       _shelx_res_checksum Found in CIF ......    Please Check
PLAT042_ALERT_1_G Calc. and Reported MoietyFormula Strings  Differ    Please Check
PLAT045_ALERT_1_G Calculated and Reported Z Differ by a Factor ...      0.50 Check
PLAT171_ALERT_4_G The CIF-Embedded .res File Contains EADP Records         2 Report
PLAT172_ALERT_4_G The CIF-Embedded .res File Contains DFIX Records         2 Report
PLAT176_ALERT_4_G The CIF-Embedded .res File Contains SADI Records         2 Report
PLAT187_ALERT_4_G The CIF-Embedded .res File Contains RIGU Records         1 Report
PLAT300_ALERT_4_G Atom Site Occupancy of N3         Constrained at       0.5 Check
PLAT300_ALERT_4_G Atom Site Occupancy of O3         Constrained at      0.25 Check
PLAT300_ALERT_4_G Atom Site Occupancy of C25        Constrained at      0.25 Check
PLAT300_ALERT_4_G Atom Site Occupancy of C26        Constrained at      0.25 Check
PLAT300_ALERT_4_G Atom Site Occupancy of C27        Constrained at      0.25 Check
PLAT300_ALERT_4_G Atom Site Occupancy of H25        Constrained at      0.25 Check
PLAT300_ALERT_4_G Atom Site Occupancy of H26A       Constrained at      0.25 Check
PLAT300_ALERT_4_G Atom Site Occupancy of H26B       Constrained at      0.25 Check
PLAT300_ALERT_4_G Atom Site Occupancy of H26C       Constrained at      0.25 Check
PLAT300_ALERT_4_G Atom Site Occupancy of H27A       Constrained at      0.25 Check
PLAT300_ALERT_4_G Atom Site Occupancy of H27B       Constrained at      0.25 Check
PLAT300_ALERT_4_G Atom Site Occupancy of H27C       Constrained at      0.25 Check
PLAT300_ALERT_4_G Atom Site Occupancy of H27D       Constrained at      0.25 Check
PLAT301_ALERT_3_G Main Residue  Disorder ..............(Resd  1  )       28% Note
PLAT302_ALERT_4_G Anion/Solvent/Minor-Residue Disorder (Resd  2  )      100% Note
PLAT304_ALERT_4_G Non-Integer Number of Atoms in ...... Resd  2         6.50 Check
PLAT721_ALERT_1_G Bond    Calc     0.97000, Rep     0.95990 Dev...      0.01 Ang.
              C24X     -H24D            1.555   1.555 ........     #   68 Check
PLAT779_ALERT_4_G Suspect or Irrelevant (Bond) Angle in CIF .... #       120 Check
              C25  -O3   -H27C    1.555   1.555   3.566            10.00 Deg.
PLAT779_ALERT_4_G Suspect or Irrelevant (Bond) Angle in CIF .... #       125 Check
              C27  -O3   -H27B    3.566   1.555   3.566            40.00 Deg.
PLAT779_ALERT_4_G Suspect or Irrelevant (Bond) Angle in CIF .... #       126 Check
```

```
              C27  -O3   -H27D    3.566   1.555   3.566             38.00 Deg.
PLAT779_ALERT_4_G Suspect or Irrelevant (Bond) Angle in CIF .... #        149 Check
              O3   -C25  -H27C    1.555   1.555   3.566              4.00 Deg.
PLAT779_ALERT_4_G Suspect or Irrelevant (Bond) Angle in CIF .... #        154 Check
              H25  -C25  -H26C    1.555   1.555   3.566             22.60 Deg.
PLAT779_ALERT_4_G Suspect or Irrelevant (Bond) Angle in CIF .... #        157 Check
              C26  -C25  -H26C    3.566   1.555   3.566             40.00 Deg.
PLAT779_ALERT_4_G Suspect or Irrelevant (Bond) Angle in CIF .... #        180 Check
              H26C -C26  -H25     1.555   1.555   3.566             14.50 Deg.
PLAT779_ALERT_4_G Suspect or Irrelevant (Bond) Angle in CIF .... #        186 Check
              C27  -C26  -H27A    3.566   1.555   3.566             33.80 Deg.
PLAT779_ALERT_4_G Suspect or Irrelevant (Bond) Angle in CIF .... #        188 Check
              O3   -C27  -H27C    3.566   1.555   1.555             19.00 Deg.
PLAT794_ALERT_5_G Tentative Bond Valency for Pt1       (II)      .       2.33 Info
PLAT811_ALERT_5_G No ADDSYM Analysis: Too Many Excluded Atoms ....          ! Info
PLAT860_ALERT_3_G Number of Least-Squares Restraints .............          9 Note
PLAT909_ALERT_3_G Percentage of I>2sig(I) Data at Theta(Max) Still        55% Note
PLAT910_ALERT_3_G Missing # of FCF Reflection(s) Below Theta(Min).          2 Note
PLAT933_ALERT_2_G Number of OMIT Records in Embedded .res File ...         15 Note
PLAT978_ALERT_2_G Number C-C Bonds with Positive Residual Density.          3 Info
```

---

   2 **ALERT level A** = Most likely a serious problem - resolve or explain
   2 **ALERT level B** = A potentially serious problem, consider carefully
  10 **ALERT level C** = Check. Ensure it is not caused by an omission or oversight
  42 **ALERT level G** = General information/check it is not something unexpected

```
   4 ALERT type 1 CIF construction/syntax error, inconsistent or missing data
  14 ALERT type 2 Indicator that the structure model may be wrong or deficient
   8 ALERT type 3 Indicator that the structure quality may be low
  28 ALERT type 4 Improvement, methodology, query or suggestion
   2 ALERT type 5 Informative message, check
```

---

checkCIF publication errors

Alert level A

```
PUBL004_ALERT_1_A  The contact author's name and address are missing,
            _publ_contact_author_name and _publ_contact_author_address.
PUBL005_ALERT_1_A  _publ_contact_author_email, _publ_contact_author_fax and
            _publ_contact_author_phone are all missing.
            At least one of these should be present.
PUBL006_ALERT_1_A  _publ_requested_journal is missing
            e.g. 'Acta Crystallographica Section C'
PUBL008_ALERT_1_A  _publ_section_title is missing. Title of paper.
PUBL009_ALERT_1_A  _publ_author_name is missing. List of author(s) name(s).
PUBL010_ALERT_1_A  _publ_author_address is missing. Author(s) address(es).
PUBL012_ALERT_1_A  _publ_section_abstract is missing.
            Abstract of paper in English.
```

---

   7 **ALERT level A** = Data missing that is essential or data in wrong format
   0 **ALERT level G** = General alerts. Data that may be required is missing

---

Publication of your CIF

You should attempt to resolve as many as possible of the alerts in all categories. Often the minor alerts point to easily fixed oversights, errors and omissions in your CIF or refinement strategy, so attention to these fine details can be worthwhile. In order to resolve some of the more serious problems it may be necessary to carry out additional measurements or structure refinements. However, the nature of your study may justify the reported deviations from journal submission requirements and the more serious of these should be commented upon in the discussion or experimental section of a paper or in the "special_details" fields of the CIF. *checkCIF* was carefully designed to identify outliers and unusual parameters, but every test has its limitations and alerts that are not important in a particular case may appear. Conversely, the absence of alerts does not guarantee there are no aspects of the results needing attention. It is up to the individual to critically assess their own results and, if necessary, seek expert advice.

If level A alerts remain, which you believe to be justified deviations, and you intend to submit this CIF for publication in a journal, you should additionally insert an explanation in your CIF using the Validation Reply Form (VRF) below. This will allow your explanation to be considered as part of the review process.

Validation response form

Please find below a validation response form (VRF) that can be filled in and pasted into your CIF.

```
# start Validation Reply Form
_vrf_PUBL004_GLOBAL
;
PROBLEM: The contact author’s name and address are missing,
RESPONSE: ...
;
_vrf_PUBL005_GLOBAL
;
PROBLEM: _publ_contact_author_email, _publ_contact_author_fax and
RESPONSE: ...
;
_vrf_PUBL006_GLOBAL
;
PROBLEM: _publ_requested_journal is missing
RESPONSE: ...
;
_vrf_PUBL008_GLOBAL
;
PROBLEM: _publ_section_title is missing. Title of paper.
RESPONSE: ...
;
_vrf_PUBL009_GLOBAL
;
PROBLEM: _publ_author_name is missing. List of author(s) name(s).
RESPONSE: ...
;
_vrf_PUBL010_GLOBAL
;
PROBLEM: _publ_author_address is missing. Author(s) address(es).
RESPONSE: ...
;
_vrf_PUBL012_GLOBAL
;
PROBLEM: _publ_section_abstract is missing.
```

```
RESPONSE: ...
;
_vrf_PLAT770_shi_3848_mz
;
PROBLEM: Suspect C-H Bond in CIF:  C26      --H27A      .       1.44 Ang.
RESPONSE: ...
;
_vrf_PLAT772_shi_3848_mz
;
PROBLEM: Suspect O-H Bond in CIF: O3       --H27D      ..       1.52 Ang.
RESPONSE: ...
;
# end Validation Reply Form
```

If you wish to submit your CIF for publication in Acta Crystallographica Section C or E, you should upload your CIF via the web. If you wish to submit your CIF for publication in IUCrData you should upload your CIF via the web. If your CIF is to form part of a submission to another IUCr journal, you will be asked, either during electronic submission or by the Co-editor handling your paper, to upload your CIF via our web site.

PLATON version of 19/10/2018; check.def file version of 15/10/2018

Datablock shi_3848_mz - ellipsoid plot

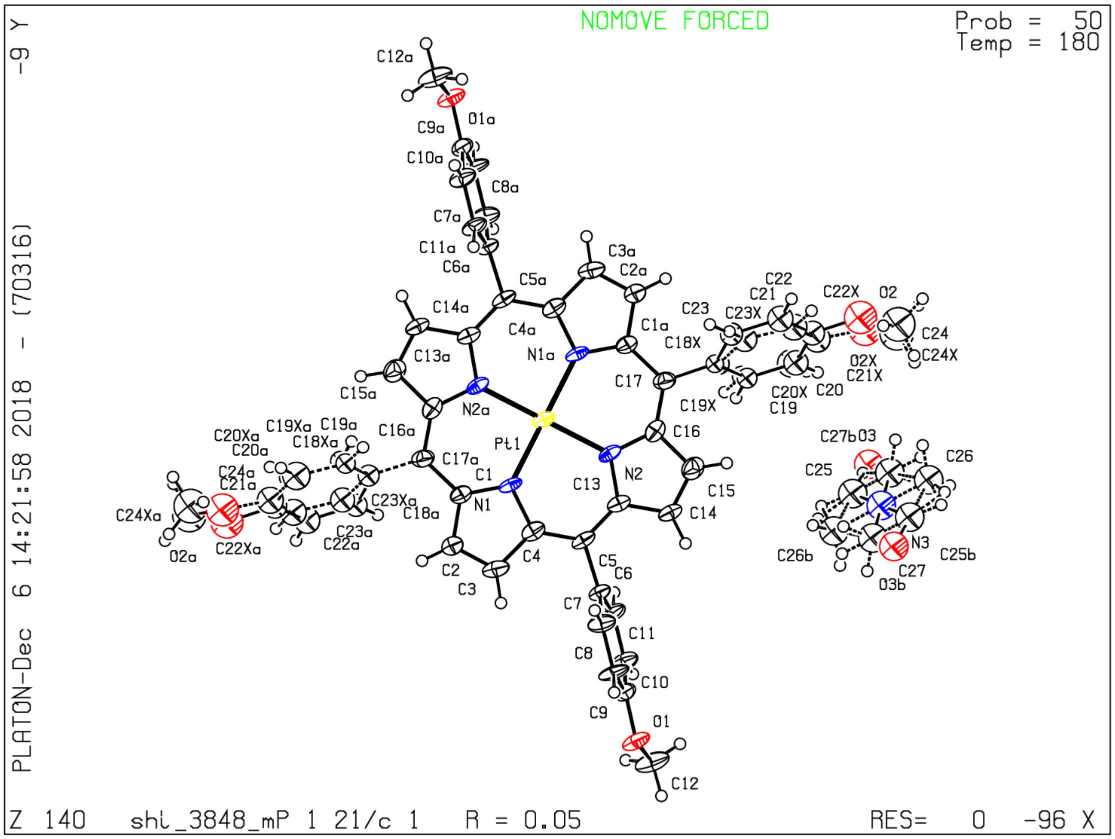

Supplement: Supplementary file 1 [file ijms-20-00710-s001.zip › Shi_3848_MZ_cifreport.pdf]
