# Supplementary material for: X-ray Structure Elucidation of a Pt-Metalloporphyrin and Its Application for Obtaining Sensitive AuNPs-Plasmonic Hybrids Capable of Detecting Triiodide Anions
Source: Int J Mol Sci. 2019 Feb 7;20(3):710. doi: 10.3390/ijms20030710 (PMC6387073; doi:10.3390/ijms20030710)
Supplement: Supplementary file 1 [file ijms-20-00710-s001.zip › Supporting Information.pdf]

Supporting Information For:

X-ray structure elucidation of Pt-metalloporphyrin and its application for obtaining sensitive nano-Au-plasmonic hybrids capable to detect iodide anions

Eugenia Fagadar-Cosma[1]*, Anca Lascu[1], Sergiu Shova[2], Mirela-Fernanda Zaltariov[2], Mihaela Birdeanu[3], Lilia Croitor[1], Adriana Balan[4], Diana Anghel[1] and Serban Stamatin[4]

[1] Institute of Chemistry Timisoara of Romanian Academy, Mihai Viteazu Ave. No. 24, Timisoara, 300223, Romania; efagadar@yahoo.com, ancalascu@yahoo.com, croitor.lilia@gmail.com, dianaracanel@yahoo.com

[2] "Petru Poni" Institute of Macromolecular Chemistry, Laboratory of Inorganic Polymers, Aleea Grigore Ghica Voda No. 41A, RO-700487 Iasi, Romania; shova@icmpp.ro, zaltariov.mirela@icmpp.ro

[3] National Institute for Research and Development in Electrochemistry and Condensed Matter, P. Andronescu Street, No. 1, 300224- Timisoara, Romania, mihaione2002@yahoo.com.

[4] University of Bucharest, Faculty of Physics - 3Nano-SAE research Center, Atomistilor Street, No 405, Măgurele, 077125, Romania, andronie@3nanosae.org, serban@3nanosae.org

* Correspondence: efagadar@yahoo.com; Tel.: +40-256-491818;

Table S1. Bond distances (Å) and angles (°).

Pt1-N1	2.000(7)	C8-C9	1.389(11)
Pt1-N2	2.017(7)	C9-C10	1.388(10)
O1-C9	1.366(8)	C10-C11	1.377(11)
O1-C12	1.425(9)	C13-C14	1.410(12)
O2-C21	1.376(10)	C14-C15	1.349(12)
O2-C24	1.421(12)	C15-C16	1.440(11)
N1-C1	1.375(11)	C16-C17	1.392(11)
N1-C4	1.401(10)	C17-C18	1.448(12)
N2-C13	1.387(10)	C22-C21	1.39
N2-C16	1.376(11)	C22-C23	1.39
C1-C2	1.417(11)	C21-C20	1.39
C1-C17[1]	1.419(11)	C20-C19	1.39
C2-C3	1.354(12)	C19-C18	1.39
C3-C4	1.453(13)	C18-C23	1.39
C4-C5	1.388(12)	O3-C25	1.22(5)
C5-C6	1.484(10)	N3-C252	1.391(10)
C5-C13	1.381(12)	N3-C25	1.391(10)
C6-C7	1.384(10)	N3-C26	1.467(10)

C6-C11	1.374(10)	N3-C27	1.462(10)
C7-C8	1.382(11)		

N1-Pt1-N2	90.0(3)	C10-C9-C8	118.8(7)
C9-O1-C12	116.7(6)	C11-C10-C9	119.9(8)
C21-O2-C24	115.2(17)	C6-C11-C10	122.4(8)
C1-N1-Pt1	128.0(5)	N2-C13-C14	110.1(8)
C1-N1-C4	105.6(7)	C5-C13-N2	125.6(8)
C4-N1-Pt1	126.4(6)	C5-C13-C14	124.2(7)
C13-N2-Pt1	127.3(6)	C15-C14-C13	107.3(8)
C16-N2-Pt1	126.7(5)	C14-C15-C16	107.7(9)
C16-N2-C13	106.0(7)	N2-C16-C15	108.7(8)
N1-C1-C2	110.7(7)	N2-C16-C17	126.8(8)
N1-C1-C17[1]	125.2(8)	C17-C16-C15	124.4(9)
C2-C1-C17[1]	124.0(9)	C11-C17-C18	117.7(8)
C3-C2-C1	107.9(9)	C16-C17-C11	123.2(9
C2-C3-C4	106.8(7)	C16-C17-C18	118.9(8)
N1-C4-C3	108.8(8)	C21-C22-C23	120
C5-C4-N1	126.3(8)	O2-C21-C22	115.5(9)
C5-C4-C3	124.8(8)	O2-C21-C20	124.4(9)
C4-C5-C6	117.4(8)	C22-C21-C20	120
C13-C5-C4	124.2(7)	C21-C20-C19	120
C13-C5-C6	118.4(7)	C18-C19-C20	120
C7-C6-C5	122.6(7)	C19-C18-C17	118.0(7)
C11-C6-C5	120.5(7)	C19-C18-C23	120
C11-C6-C7	116.9(7)	C23-C18-C17	122.0(7)
C8-C7-C6	122.3(8)	C18-C23-C22	120
C7-C8-C9	119.6(7)	C25-N3-C26	117(2)
O1-C9-C8	115.6(7)	C27-N3-C26	109(2)
O1-C9-C10	125.5(8)	O3-C25-N3	120(4)

Symmetry code: [i)] 1-*x*, 1-*y*, 2-*z*
